# Supplementary material for: Effect of Two Nutritional Strategies to Balance Energy and Protein Supply in Fattening Heifers on Performance, Ruminal Metabolism, and Carcass Characteristics
Source: Animals (Basel). 2020 May 14;10(5):852. doi: 10.3390/ani10050852 (PMC7278469; doi:10.3390/ani10050852)
Supplement: Supplementary file 1 [file animals-10-00852-s001.pdf]

animals

Article

Effect of Two Nutritional Strategies to Balance Energy and Protein Supply in Fattening Heifers on Performance, Ruminal Metabolism, and Carcass Characteristics

Rodrigo A. Arias [1,2,*], Gonzalo Guajardo [3], Stefan Kunick [3], Christian Alvarado-Gilis [1] and Juan Pablo Keim [1]

Table S1. Values of the *in situ* degradation characteristics of OM and CP.

Variable	Beet Pulp	Grass Silage	Canola Meal	Triticale
OM				
A	12.7	31.8	20.9	12.72
B	85.4	57.3	62.2	82.5.95
C	0.141	0.096	0.203	0.447
PD	98.6	89.6	83.1	92.3
CP				
A	8.8	44.3	12.2	2.6
B	86.7	34.4	79.4	89
C	0.132	0.165	0.234	0.562
PD	95.6	78.1	91.5	91.5

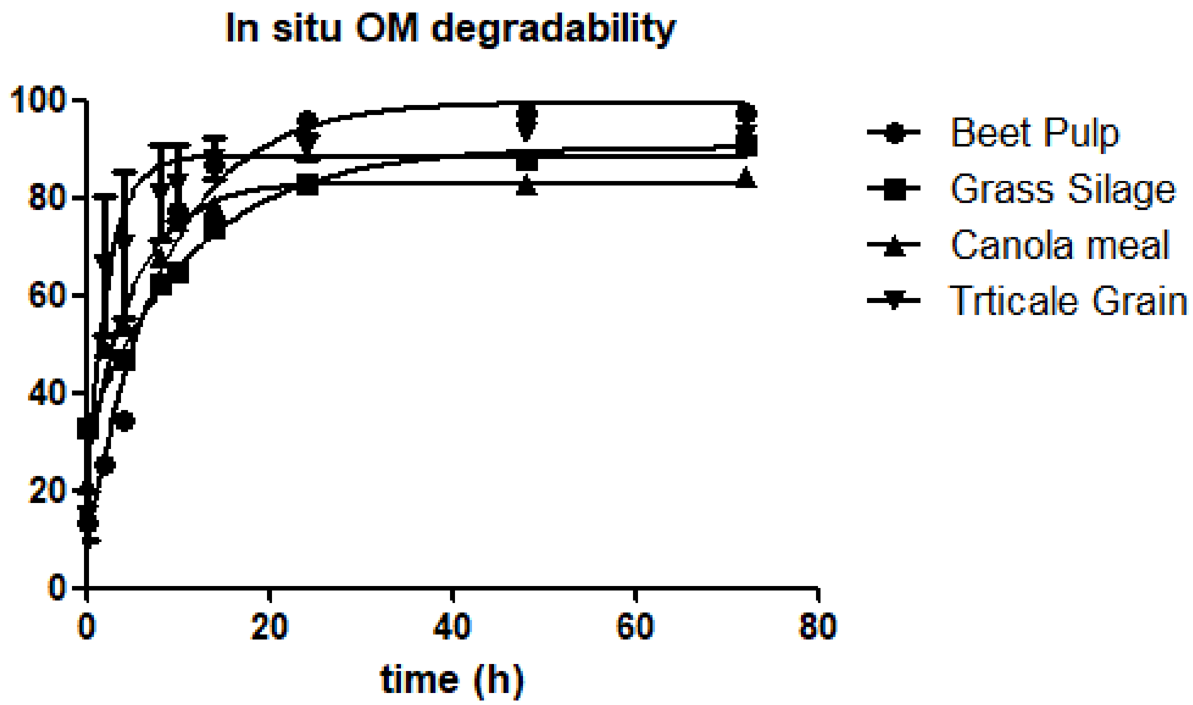

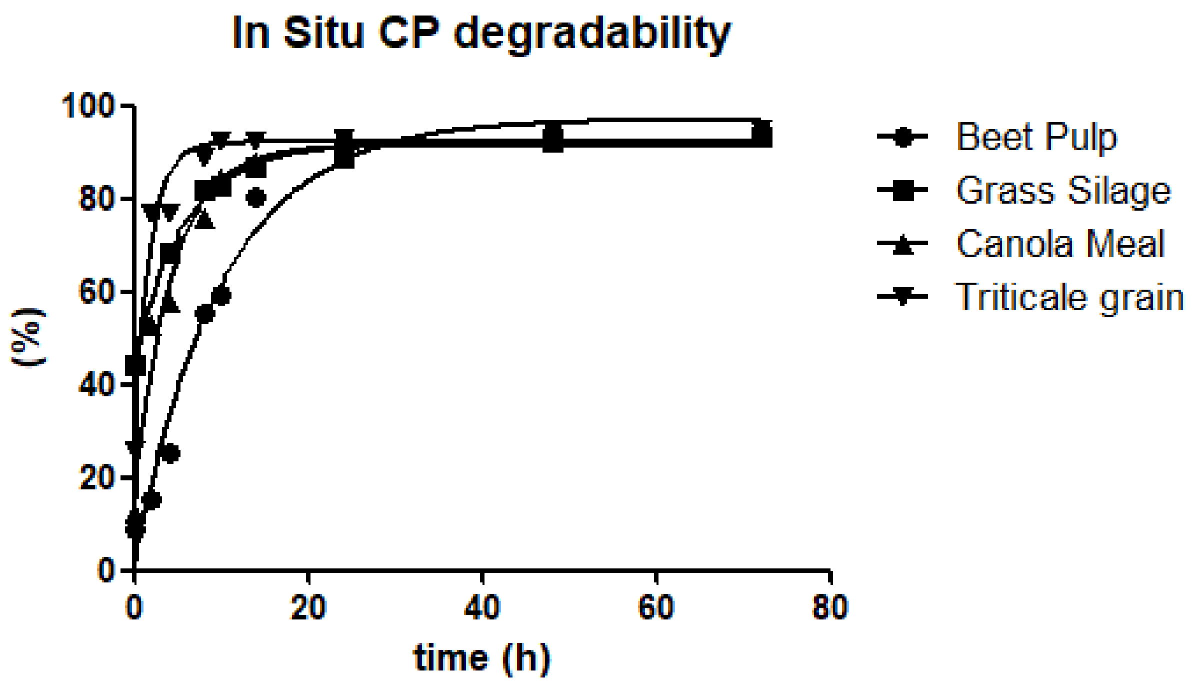

Figure S1. Adjusted degradation curves of the OM and CP.
